# Probiotic-Based Bacteriocin: Immunity Supplementation Against Viruses. An Updated Review

**DOI:** 10.3389/fmicb.2022.876058

**Published:** 2022-07-26

**Authors:** Muhammad Umair, Saqib Jabbar, Lu Zhaoxin, Zhang Jianhao, Muhammad Abid, Kashif-Ur R. Khan, Sameh A. Korma, Mashail A. Alghamdi, Mohamed T. El-Saadony, Mohamed E. Abd El-Hack, Ilaria Cacciotti, Synan F. AbuQamar, Khaled A. El-Tarabily, Liqing Zhao

**Affiliations:** ^1^Department of Food Science and Engineering, College of Chemistry and Environmental Engineering, Shenzhen University, Shenzhen, China; ^2^Key Laboratory of Optoelectronic Devices and Systems, College of Physics and Optoelectronic Engineering, Ministry of Education and Guangdong Province, Shenzhen University, Shenzhen, China; ^3^Food Science Research Institute (FSRI), National Agricultural Research Centre (NARC), Islamabad, Pakistan; ^4^College of Food Science and Technology, Nanjing Agricultural University, Nanjing, China; ^5^Institute of Food and Nutritional Sciences, Pir Mehr Ali Shah Arid Agriculture University, Rawalpindi, Pakistan; ^6^Department of Pharmaceutical Chemistry, Faculty of Pharmacy, The Islamia University of Bahawalpur, Bahawalpur, Pakistan; ^7^Department of Food Science, Faculty of Agriculture, Zagazig University, Zagazig, Egypt; ^8^Department of Biology, Faculty of Science, King Abdulaziz University, Jeddah, Saudi Arabia; ^9^Department of Agricultural Microbiology, Faculty of Agriculture, Zagazig University, Zagazig, Egypt; ^10^Department of Poultry, Faculty of Agriculture, Zagazig University, Zagazig, Egypt; ^11^Department of Engineering, INSTM RU, University of Rome “Niccolò Cusano”, Rome, Italy; ^12^Department of Biology, College of Science, United Arab Emirates University, Al-Ain, United Arab Emirates; ^13^Khalifa Center for Genetic Engineering and Biotechnology, United Arab Emirates University, Al-Ain, United Arab Emirates; ^14^Harry Butler Institute, Murdoch University, Murdoch, WA, Australia

**Keywords:** antiviral immunity, bacteriocin, immune interaction, immunomodulatory, probiotics, viral infection

## Abstract

Viral infections are a major cause of severe, fatal diseases worldwide. Recently, these infections have increased due to demanding contextual circumstances, such as environmental changes, increased migration of people and product distribution, rapid demographic changes, and outbreaks of novel viruses, including the COVID-19 outbreak. Internal variables that influence viral immunity have received attention along with these external causes to avert such novel viral outbreaks. The gastrointestinal microbiome (GIM), particularly the present probiotics, plays a vital role in the host immune system by mediating host protective immunity and acting as an immune regulator. Bacteriocins possess numerous health benefits and exhibit antagonistic activity against enteric pathogens and immunobiotics, thereby inhibiting viral infections. Moreover, disrupting the homeostasis of the GIM/host immune system negatively affects viral immunity. The interactions between bacteriocins and infectious viruses, particularly in COVID-19, through improved host immunity and physiology are complex and have not yet been studied, although several studies have proven that bacteriocins influence the outcomes of viral infections. However, the complex transmission to the affected sites and siRNA defense against nuclease digestion lead to challenging clinical trials. Additionally, bacteriocins are well known for their biofunctional properties and underlying mechanisms in the treatment of bacterial and fungal infections. However, few studies have shown the role of probiotics-derived bacteriocin against viral infections. Thus, based on the results of the previous studies, this review lays out a road map for future studies on bacteriocins for treating viral infections.

## Introduction

Viral infections are the primary cause of numerous diseases and deaths (Villena et al., 2020). These infections affect several tissues and organs, such as the colon (e.g., rotavirus), upper respiratory tract and lungs (e.g., rhinovirus and influenza), liver (e.g., hepatitis B virus), leukocytes [e.g., human immunodeficiency virus (HIV)], spinal cord (e.g., poliovirus), and vascular endothelial cells (e.g., Ebola; Dreyer et al., 2019; Weeks and Chikindas, 2019; Foysal et al., 2020; Jenab et al., 2020; Villena et al., 2020). Some viruses are more vulnerable to extreme conditions because of a weakened immune system. Histological examinations of patients with viral infections revealed severe destruction of tissue structure, resulting in their death (Jayawardena et al., 2020).

Previous studies have investigated various sources of antimicrobial agents from natural plants. Numerous antiviral agents have been proposed and verified as therapeutic targets in viral drugs (Muhammad et al., 2018; Umair et al., 2020, 2022; Riaz Rajoka et al., 2021; Senan et al., 2021; Youssef et al., 2021; Rajoka et al., 2022). Several treatments act as effective tools in viral infections, such as gene therapy; administration of pro- and anti-inflammatory cytokines, pegylated interferon-α (IFN-α), and ribavirin; recombinant human tumor necrosis factor-alpha (TNF-α) antagonist treatment; and highly active antiretroviral therapy, which includes multiple antiretroviral agents; or a combination of these therapies (Lehtoranta et al., 2020; Lopez-Santamarina et al., 2020). However, viral genome mutations are lethal for viral replication and cause viral resistance to the adopted antiviral therapies. Therefore, it is necessary to provide other preventive or supplementary interventions.

The gastrointestinal tract (GIT) is a complex ecosystem hosting millions of resident microorganisms, known as the gut microbiota (GM; Libertucci and Young, 2019). The microbiome consists of microorganisms and their genetic material and plays an essential role in host physiology and metabolism by supplying genetic elements absent in the host genome (Hall et al., 2017). Additionally, a strong correlation exists between COVID-19 infection and the GM community (Chen et al., 2020). Further, it has been reported that patients with COVID-19 demonstrate a reduced number of *Lactobacillus* and *Bifidobacterium* due to intestinal microbial dysbiosis (Chen et al., 2020).

In particular, when probiotics, the “cultured” (living) microorganisms beneficial to the host, are ingested in adequate amounts (Quilodrán-Vega et al., 2020; El-Saadony et al., 2021) as a compliment or element inherent in food, they principally improve the GM composition (Minj et al., 2021), treat dysbiosis, and prevent viral diseases (Hill et al., 2014). Notably, bacteriocins are antimicrobial peptides produced by significant bacterial cell lines (Khalil et al., 2022; Le Morvan De Sequeira et al., 2022; Naseef et al., 2022). Additionally, these bacteriocins modulate the systemic immune and mucosal systems of humans and animals, protecting them from several viral infections (Alvarez-Vieites et al., 2020).

However, the interactions between viral diseases and the immunity induced by probiotics remain unknown, including the mechanism of the reported antiviral activity of probiotics. Moreover, proteolytic enzymes degrade bacteriocins, causing their instability in different body parts, such as the GIT, serum, liver, and kidneys, thus limiting their industrial applications (Weeks and Chikindas, 2019). Given these considerations, the present review provides a comprehensive overview of the role of the probiotics-based bacteriocin as an immune-modulating agent in combating viral infections, its stability in the GIT microbiota, and the bacteriocin mechanisms of immune modulation. Furthermore, the evolving zoonotic viruses and their animal reservoirs for viral progenitors will be described in this study, including the viral transmission route from animal to human, focusing on the most recent published studies.

## Probiotics as Immunobiotics

In general, eukaryotic hosts have a high bacterial load, predominantly habituating GIT (Bibi et al., 2021; Youssef et al., 2021; Rajoka et al., 2022). However, some of these microorganisms are currently susceptible to pathogenesis (Round and Mazmanian, 2009). Therapeutic antibiotics occasionally disrupt the intestinal microbiota, which subsequently initiate microbial pathogenesis (Foysal et al., 2020).

Probiotics are heat- and pH-stable, colorless, odorless, and tasteless, which indicate their suitability in food applications. Lactic acid bacteria (LAB) and bifidobacteria are the most commonly used probiotic bacteria. LAB essentially preserves the integrity of the human gut wall and maintains a healthy microbiome, including the inhibition of gut pathogen proliferation. Other genera, such as enterococci, and yeast, such as *Saccharomyces*, have been proposed and exploited as probiotic agents (Ou et al., 2019; Jaffar and Zailan, 2021; Lai et al., 2021; Liu et al., 2022).

**Figure 1:**
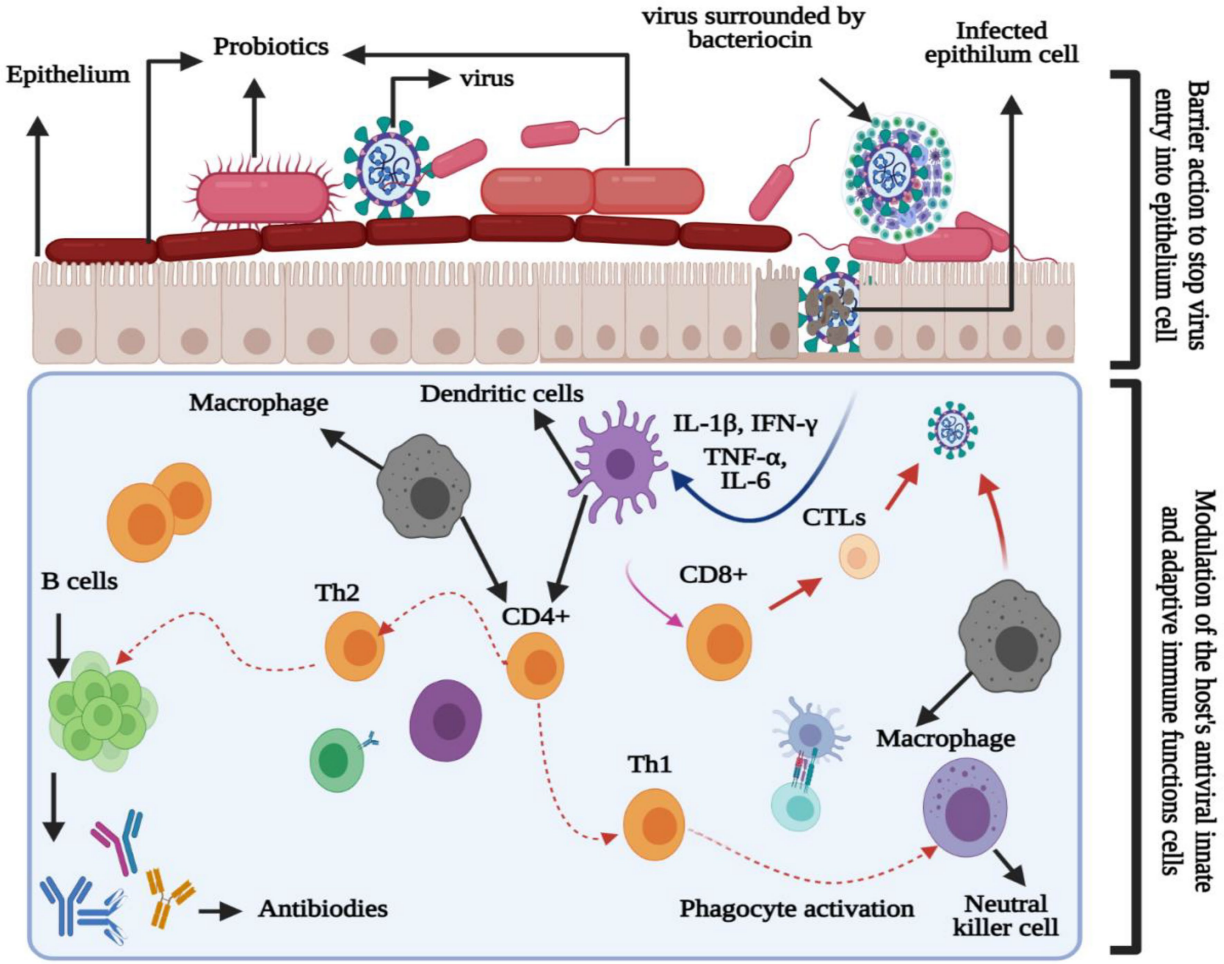
Immunomodulatory properties of probiotics for enhancing phagocytosis against the virus.

**Figure 2:**
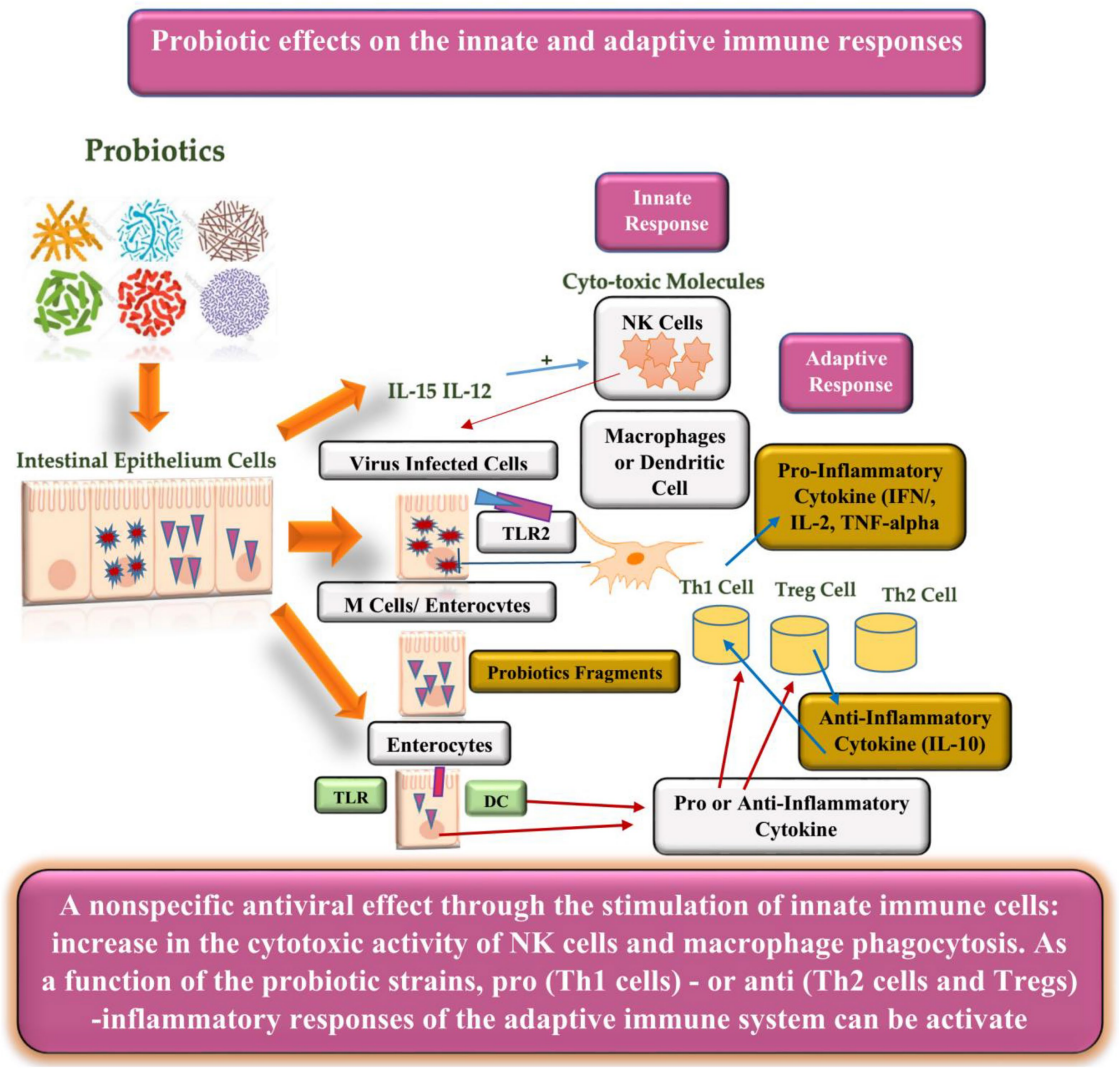
Probiotics and innate and adaptive immune interaction against viral infections.

Probiotics have the potential for lowering lactose and cholesterol levels, cancer prevention, and lowering the risk of secondary infections (Li et al., 2021). Another benefit of probiotics is their immunobiotic nature in modulating or enhancing the immune (mucosal) system. As viruses are in direct contact with the mucosal (respiratory, genital, or gastrointestinal) surfaces, they must overcome three lines of defense: (1) the mucus coat, (2) innate, and (3) adaptive immune responses. Therefore, probiotics interfere with the viruses directly or indirectly to improve their phagocytosis (Alvarez-Vieites et al., 2020). The direct response can be subcategorized into two stages (Figure 1): barrier activity toward viral particles before entering the epithelial cells and the modulation of the host antiviral immune response. Moreover, the barrier activity toward viruses includes (1) enhanced mucosal barrier activity, (2) direct probiotic-virus association, (3) secretion of antiviral inhibitory metabolites (bacteriocins), and (4) inhibition of viral attachment to host cells (Belguesmia et al., 2020). Additionally, the effects of probiotic modulation on the immune cells can be observed in lymphocytes, hematopoietic stem cells, T cells, macrophages, natural killer cells, and dendritic cells (DCs; Alvarez-Vieites et al., 2020; Belguesmia et al., 2020; Li et al., 2021). Figure 2 illustrates the innate and adaptive antiviral reaction mechanisms and immunomodulatory effects of probiotics to improve viral phagocytosis (Belguesmia et al., 2020).

Probiotics indirectly interfere with the virus by (1) altering the condition of the cells, (2) enhancing or suppressing innate and adaptive immunity through the associated molecular signaling pathways, (3) protecting themselves against viral particles that compete for cell-surface adhesion, (4) reducing inflammatory processes by controlling innate immunity through Toll-like receptors and other signaling pathways, and (5) producing antiadhesive substances against the viruses. Thus, probiotics prevent its adhesion to the host cell receptor by binding to the receptor of the invading virus (Abdolalipour et al., 2020; Alvarez-Vieites et al., 2020; Villena et al., 2020).

Cell adhesion is a multistage process classified into specific and nonspecific adhesion. Nonspecific adhesion occurs when a probiotic enters the cell and is mainly governed by physicochemical characteristics, such as contacts or hydrogen bonds (Kumar, 2021; Wang et al., 2021b). Specific adhesion involves the interaction between probiotic adhesins and their epithelial receptors, which is an irreversible connection that is more potent than nonspecific adhesion (Cao et al., 2020; Hwang et al., 2022). The adhesion capacities of the selected strains were determined by examining their bacterial cell-surface features in a Caco-2 cell model, which is comparable in morphology to the intestinal epithelium. The composition of the microbiota profoundly impacts human health, and diet is a critical factor in determining its composition (Cao et al., 2020; Kumar, 2021; Wang et al., 2021b; Hwang et al., 2022).

A few strategies for modulating the host immune response, such as increasing essential nutrient intake and developing potential functional foods, are becoming popular to influence the activity of immune cells (Foysal et al., 2020). Dietary supplementation with probiotics enhances the states of the intestine, liver, and the immune system as well as the structure and functions of genetically modified substances. Probiotics considerably enhance the immune-associated metabolic pathways, such as amino sugar pathway, nucleotide sugar pathway, interleukin 17 (IL-17) signaling, and quorum sensing (Alvarez-Sieiro et al., 2016). In contrast, the absence of probiotics prevents glyoxylate and dicarboxylate metabolism. Bacterial communication *via* extracellular diffusible signaling molecules (quorum sensing) enables bacteria to coordinate their group activity and multicellular functioning. Bacteriocins also operate as signaling peptides, communicating with other bacteria through quorum sensing and bacterial cross-talk within microbial communities or with host immune cells (Foysal et al., 2020). The (N-acyl) homoserine lactone is used as a signal molecule in gram-negative bacteria, whereas peptides, including several bacteriocins, are frequently used as signaling molecules in gram-positive bacteria (Drider et al., 2016). In general, peptide-based quorum sensing in gram-positive bacteria mediates a two-component regulatory signal transduction system, comprising a membrane-bound histidine protein kinase (HPK) and an intracellular response regulator (RR) (Drider et al., 2016; Foysal et al., 2020). Thus, it has been postulated that few bacteriocins act as inhibitors at high concentrations and signaling molecules at low concentrations (Alvarez-Sieiro et al., 2016; Drider et al., 2016; Foysal et al., 2020).

HPK phosphorylates RR, which causes a response at the transcriptional level. The autoinducing peptide primarily serves as a signaling molecule; however, some autoinducing peptides also act as antimicrobials (Prazdnova et al., 2022). Bacteriocin nisin is the predominant example of this dual functioning. Nisin acts as a killer and signaling molecule and stimulates its production in a density-dependent manner (Belguesmia et al., 2020). Thus, probiotic consumption is the only nutritional practice capable of lowering the risk of viral infection and enhancing human immune responses (Belguesmia et al., 2020; Chen et al., 2020; Prazdnova et al., 2022). Following these considerations and experimental reports, it is crucial to discover new strategies for preventing or reducing viral infections to minimize virus-related mortality, morbidity, and economic losses.

## Bacteriocin Stability and Mode of Action

Bacteriocins are multifunctional proteinaceous compounds synthesized from ribosomal RNA with antimicrobial potential against pathogens at definite concentrations (Belguesmia et al., 2020), demonstrating their biotechnological capability (Chikindas et al., 2018). Bacteriocins are classified into three groups according to their physicochemical and structural features: classes I, II, and III (Chikindas et al., 2018). Moreover, during the early stages of development, bacteriocins are only active against closely related bacteria. However, the mode of action of bacteriocins against viruses is not yet fully understood. According to the available reports, bacteriocins exhibit two mechanisms for combating viral infection (Wachsman et al., 2003).

The first mechanism involves preventing viral particle aggregation and blocking the sites of host cell receptors (Wachsman et al., 2003). Additionally, certain bacteriocins possess an antiviral activity that inhibits viral penetration into human cells. For instance, it was shown that duramycin, a class I bacteriocin, blocks the Zika virus coreceptor TIM1 and subsequently inhibits its entry (Tabata et al., 2016). In the second mechanism, several bacteriocins induce cytopathic effect and reduce viral release without interfering with viral entry (Tabata et al., 2016). The mechanisms of bacteriocins against viral infection are related to their interaction with the late steps of the viral cycle, which strongly influence the main reactions of the viral multiplication step (Tabata et al., 2016). Additionally, within these bacteriocins, those recognized as antiviral molecules can bind to the lipid membranes of enveloped viruses as they are hydrophobic. Thus, probiotics exhibit their inhibitory action by interfering with cellular and viral membrane fusion (Cavicchioli et al., 2018). Given these considerations, the inactivity of several bacteriocins against nonenveloped viruses is attributed to the structural differences compared with active bacteriocins, particularly to the lack of hydrophobicity (Badani et al., 2014; Cavicchioli et al., 2018).

Bacteria produce bacteriocins as a defense or contact signal (Chikindas et al., 2018). A previous report indicated that the leading interface site among the host immune system, microorganisms in the GIT, and GM bacterial colonization influences the development of the adaptive immune system (Round and Mazmanian, 2009). Bacteriocins also elicit an immune response, causing changes in the GIT population. Another study investigated the potential of bacteriocins in penetrating the gut–blood barrier (i.e., Caco-2), which is further dependent on the physicochemical and biochemical characteristics of the membrane (Dreyer et al., 2019).

Numerous bacteriocins have been demonstrated to be effective against viral infections (Dobson et al., 2012). However, the route of the bacteriocin administration impacts the proteolytic enzyme activity. For instance, bacteriocins, such as nisin F, were active against pathogens when injected into the peritoneal cavity of mice. However, nisin F acts as a growth stabilizer for GM (Van Staden et al., 2011). It also plays a role in GIT colonization, promoting bacteriocin-producing strains (Kommineni et al., 2015). Thus, inducing bacteriocin-producing strains in the GIT is a viable alternative for monitoring viral infections, drug resistance, enteric invasion, and pathogen dissemination in the GIT (Hegarty et al., 2016).

Additionally, several factors influence the bacteriocin sensitivity of the target bacterium, including the presence of membrane-disrupting neutralizing molecules and physicochemical properties of the environment (ionic strength, pH). However, using bacteriocin is not similar to regular antibodies (Weeks and Chikindas, 2019); the microbial immunity against bacteriocin is extremely rare compared with that against antibodies (De Freire Bastos et al., 2015). Furthermore, microbial strains that induce resistance possess different mechanisms of antibody resistance (Chatterjee et al., 2005). For instance, microbial species undergo major structural modifications, such as changing the phospholipid and fatty acid composition of the cell membrane, increasing the galactose and D-alanyl ester content of the cell wall, or forming a thicker cell wall to avert bacteriocin docking with lipid II, thus developing bacteriocin resistance (Crandall and Montville, 1998; Chatterjee et al., 2005). Additionally, other microbial species possess some structural modifications that involve forming a requirement for Mn^2+^, Mg^2+^, Ca^2+^, and Ba^2+^ in the cell wall or membrane (Crandall and Montville, 1998; Chatterjee et al., 2005).

Besides these structural changes, nonstructural modifications are also involved, which are capable of inducing bacteriocin resistance, trigger and induce mutation in the bacteriocin susceptibility-associated sensor (Collins et al., 2012), trigger the inactivation of the gene encoding bacterial RNA polymerase, produce nonproteolytic bacteriocins capable of inactivating enzyme-induced dehydroalanine-level reduction (Jarvis and Farr, 1971), and produce pH-related resistance alterations (Hasper et al., 2006). However, the molecular size, hydrophobicity, migration intensity, charge used to cross the epithelial cell membrane, and capacity to re-enter the tissue cells are the properties of bacteriocins that need further investigation. In this regard, several recently published studies on the immunomodulatory properties of bacteriocins have been noted (Jenab et al., 2020).

## Bacteriocin as a Potential Immunobiotic Agent for Viral Infections

Reactions to immunomodulatory therapies are an emerging strategy against viral infection. Researchers studied that enhancing the body’s immune system by employing probiotic-based bacteriocin helps in fighting against viral diseases (Table 1; Yan et al., 2020, 2022; Wang et al., 2021a; Liu et al., 2022; Zhang et al., 2022). Jayawardena and coworkers also reported that bacteriocin modulates the immune system and prevents viral infections, such as COVID-19 (Jayawardena et al., 2020).

**Table 1:** Antiviral/antimicrobial activity of different bacteriocin as potential immunomodulatory agent and their action mode against viral disease.

**Compound**	**Role**	**Mode of action**	**Findings**	**References**
Bacteriocin	Shows immunomodulatory properties		GIT integrity, immune function is critical for preventing and controlling viruses	Belguesmia et al., 2020
Cationic antimicrobial peptides	Shows immunomodulatory properties	Hydrophobicity, positive charge, and small size immune system	Interferes and stimulates immune system	Sahl et al., 2005
Bacteriocin	Shows immunomodulatory properties	Changes in DCs, improves activities of T and B lymphocytes, monocytes, and macrophages, the IFN, and interleukin development	Enhances viral phagocytosis	Soltani et al., 2021
Bacteriocin	Stimulates non-specific immunity releases pro-inflammatory cytokines	TNF-α and IL-6	Increases the rotavirus-specific IgM and secretes cell and IgA responses to toxins	Soltani et al., 2021
Bacteriocin	Enhances immune responses	Reduces the PRR stimulation	Promotes the tumor necrosis factor-alpha (TNF) and the B-cell nuclear factor kappa-light-chain-enhancer	O’Callaghan et al., 2012
*Bifidobacterium bifidum*	Enhances immune responses	Mouse-adapted influenza A (H1N1) infection BALB/c model	Improves humoral and cellular immunity and reduces IL-6 activity in the lungs	Mahooti et al., 2019
*Lactobacillus delbrueckii* subsp. *bulgaricus*	Enhances immune responses	Inhibits influenza A/chicken/Germany replication of the Weybridge (H7N7) and Rostock (H7N1) strains	Shows effectiveness against influenza tested strains in mice model	Serkedjieva et al., 2000
Nisin-bateriocin	Enhances immune responses	Nisin-fed mice in the virus-infected model	Nisin has more significant immunomodulatory properties than the tested human cationic (LL-37) peptides	Kindrachuk et al., 2013
Nisin-bateriocin	Enhances immune responses	Shows antigenic response of nisin *in vivo* against viruses	Increases IL-6 and IL-10	Brand et al., 2010
Bacteriocin	Enhances immune responses	Shows anti-influenza efficacy in mouse model	Shows effectiveness against anti-influenza virus	Ermolenko et al., 2019

DCs, dendritic cells; IFN, interferons; TNF-α*, Tumour necrosis factor*α; IL-6, interleukin-6; PRRs; pattern recognition receptors IgM; IgA; IL-10, interleukin-10.

The enterocins CRL35 and ST4V have also shown bacteriocin activities against herpes simplex virus 1 (HSV-1) and HSV-2 by influencing intracellular viral proliferation and interacting with the late steps of viral replication (Wachsman et al., 2003; Todorov et al., 2005). Additionally, subtilisin A and the pediocin-like bacteriocin ST5Ha showed anti-HSV activity with a selectivity index (CC50/EC50) of 173 (Quintana et al., 2014; Soltani et al., 2021). Further, Cavicchioli et al. (2018) reported that the bacteriocins from *Enterococcus durans*, *Geo9, Ge12,* and *He17* inhibited the poliovirus (PV-1; Férir et al., 2013), revealing the dual antiviral action against HIV and HSV. They further revealed the transmission of antibiotic-derived prototype peptide containing the post-translationally transformed–special carbocyclic abionin residue. The same authors also identified labyrinthopeptin A1 (LabyA1) as a bacteriocin, emphasizing its ability to inhibit the viral cell-to-cell transfer between HIV-infected T cells and uninfected CD4 (+) T cells; moreover, it inhibits HIV capture by DC-SIGN+-cells, thereby inhibiting the transmission of the captured virus to uninfected CD4 (+) T cells (Férir et al., 2013; Table 2).

**Table 2:** Antiviral/antimicrobial activity of different probiotic based bacteriocin and their producer strains along with mode of actions.

**Bacteriocin**	**Probiotic strains**	**Antiviral/antimicrobial activity**	**Mode of action**	**Reference**
*Lactobacillus gasseri* SBT2055	*L. gasseri*	A/H1N1 and B influenza viruses	Vaccine-specific antibody production	Nishihira et al., 2018
*Lactobacillus acidophilus* IgG and HI NDV	*L. acidophilus*	A new castle virus disease	IgG and HI NDV	Gharajalar et al., 2020
*Lactobacillus plantarum* DR7	*L. plantarum*	Upper respiratory tract viral infections	Suppresses plasma proinflammatory cytokines (IFN-γ, TNF-α)	Chong et al., 2019
Enterocin CRL35	*Enterococcus faecium*	HSV-1 HSV-2	Inhibits the late-stage replication	Wachsman et al., 1999
*Lactobacillus helveticus* LZ-R-5	*L. helveticus*	Associated with the immune system	Immunostimulatory activity	You et al., 2020
*L. plantarum* C70	*L. plantarum*	Have possible bioactivities in food industries, including anticancer, antidiabetic, and antioxidant activities	Bioactivities	Ayyash et al., 2020
*L. plantarum* SP8	*L. plantarum*	Excellent biosorption ability toward methylene blue (MB)	Biosynthesis of selenium nanoparticles (SeNPs)	Li et al., 2021
*L. plantarum* SP8	*L. plantarum*	Shows specific antioxidant activity	Bioactivities	Zhang et al., 2020
EPS103	*L. plantarum* JLAU103	Shows scavenging abilities against hydroxyl, ABTS, and DPPH radicals	Bioactivities	Min et al., 2019
Bacteriocin	*Lactobacillus delbrueckii* sub sp. *bulgaricus*	Shows anti-Virus activity. A/chicken/Germany, Weybridge (H_7_N_7_), Rostock (H_7_N_1_)	Inhibits replication, glycoproteins neuraminidase	Serkedjieva et al., 2000
Enterocin ST4V	*E. faecium* ST4V	HSV-1 HSV-2	Inhibits the late-stage replication	Todorov et al., 2005
Labyrinthopeptin A1 (LabyA1)	*Actinomadura namibiensis*	Shows Anti-HIV-1 activity	Suppresses intercellular transmission between HIV-infected T cells and uninfected CD4 (+) T cells	Férir et al., 2013
LabyA1 + raltegravir	*A. namibiensis* + antiretroviral agents	Shows anti-HIV-1 activity anti-HSV-2 activity	Inhibits the transmission of HIV from DC-SIGN+ cells to uninfected CD4 (+) T cells	Al Kassaa et al., 2014
LabyA1 + LabyA2	*A. namibiensis* DSM6313	Associated with carcinoma-derived lung cells	Inhibits human respiratory syncytial virus (HRSV)	Haid et al., 2017
Cell-free supernatants (CFS)	*Lactobacillus curvatus 1*	Associated with murine norovirus (MNV)	Inhibits intracellular virus replication	Lange-Starke et al., 2014
Bacteriocin B1	*L. delbrueckii*	Anti-Virus	Inhibits intracellular virus replication	Serkedjieva et al., 2000
Bacteriocin	*Lactobacillus* spp.	Anti-HIV-1 and Anti-HSV-2	Lactic acid and hydrogen peroxide protein denaturing reactions	Conti et al., 2009
Non-protein cell wall component	*Lactobacillus brevis*	Anti-HSV-2	Reduces HSV-2 replication	Mastromarino et al., 2011
*Lactobacillus paracasei* sub sp*. rhamnosus*	*L. paracasei* sub sp. *rhamnosus*	Vesicular stomatitis viruses	Adheres to the particles	Botić et al., 2007
*L. plantarum*	*L. plantarum*	Vesicular stomatitis viruses	Adheres to the particles	Botić et al., 2007
*Lactobacillus reuteri*	*L. reuteri*	Vesicular stomatitis viruses	Adheres to the particles	Botić et al., 2007
*Enterococcus faecium* NCIMB 10415	*Enterococcus faecium* NCIMB 10415	Influenza virus H1N1	Adheres to the particles	Wang et al., 2013
*L. gasseri* CMUL57	*L. gasseri* CMUL57	HSV-2	Adheres to the particles	Al Kassaa et al., 2014
*L. plantarum* L-137	*L. plantarum* L-137	Influenza virus H1N1	Elicits a pro-inflammatory response	Maeda et al., 2009
*Lactobacillus fermentum* CECT5716	*L. fermentum* CECT5716	Influenza virus H1N1	Improves the formation of antibodies against H1N1	Olivares et al., 2007
*Lactobacillus casei* DN114-001	*L. casei* DN114-001	Influenza virus H1N1	Improves the formation of antibodies against H1N1	Boge et al., 2009
*L. gasseri* PA 16/8, *Bifidobacterium longum* SP07/3, and *Bifidobacterium bifidum* MF 20/5	*L. gasseri* PA 16/8, *B. longum* SP07/3, and *B. bifidum* MF 20/5	Common cold virus	Inhibits intracellular virus replication	De Vrese et al., 2006
*Lactobacillus rhamnosus* GG	*L. rhamnosus* GG	Respiratory virus infections	Inhibits intracellular virus replication	Rautava et al., 2008
*L. acidophilus* NCFM	*L. acidophilus* NCFM	Influenza-like symptoms	Inhibits intracellular virus replication	Leyer et al., 2009
Enterocin AAR-71	*Enterococcus faecalis*			Qureshi et al., 2006
Enterocin AAR-74	*E. faecalis*	Proliferation of coliphage HSA	Inhibits intracellular virus replication	Qureshi et al., 2006
Enterocin ST5Ha	*E. faecium*			Todorov et al., 2010
Enterocin ST4V	*Enterococcus mundtii*	Herpes viruses HSV-1 and HSV-2	Enterocins CRL35 and ST4V were used on the multiplication of virus particles	Todorov et al., 2005
Enterocin CRL35	*E. mundtii*	Inactive against herpes viruses	A derivative of enterocin CRL35, lacking two cysteine residues	Wachsman et al., 2003
Bacteriocin	*L. delbrueckii subsp. bulgaricus* 1,043	Influenza viruses	Inhibits intracellular virus replication	Serkedjieva et al., 2000

In contrast, Lange-Starke et al. (2014) reported the ineffective action of the bacteriocins produced by *Lactococcus lactis* subsp. *lactis*, such as nisin, against influenza A (H1N1), HSV-1, murine norovirus, feline herpesvirus KS285, and Newcastle disease virus Montana. Semipurified bacteriocins obtained from two LAB strains isolated from goat milk (i.e., *GLc03* and *GLc05* from *L. lactis*, and *GEn09*, *GEn12*, *GEn14*, and *GEn17* from *E. durans*) were screened in terms of cytotoxicity in Vero cells (CC50) and based on their antiviral activities against PV-1 and HVS1 (Villena et al., 2020).

A previous study showed that bacteriocins elicit an innate immune response during viral infection and induce pathogen death *via* inflammasomes (Weeks and Chikindas, 2019). The inflammasome is a multiprotein complex formed inside the cell by the nucleotide-binding oligomerization domain-like receptors (NOD-like receptors), also known as nucleotide-binding leucine-rich repeat receptors (NLRs) and DNA sensors. These inflammasomes trigger a sequence of dangerous signals elicited by pathogens and cellular stress (McNab et al., 2015). Further, another study revealed that the anti-inflammatory cytokines are inhibited *via* inflammatory signaling pathways by bacteriocin through their interaction with mitogen-activated protein kinase and nuclear factor-kappa B (Yoon and Sun, 2011). Consequently, bacteriocins regulate inflammasome activation in organisms that previously experienced inflammation due to viral infections (Dicks et al., 2018; Abdolalipour et al., 2020; Alvarez-Vieites et al., 2020; Antushevich, 2020).

**Figure 3:**
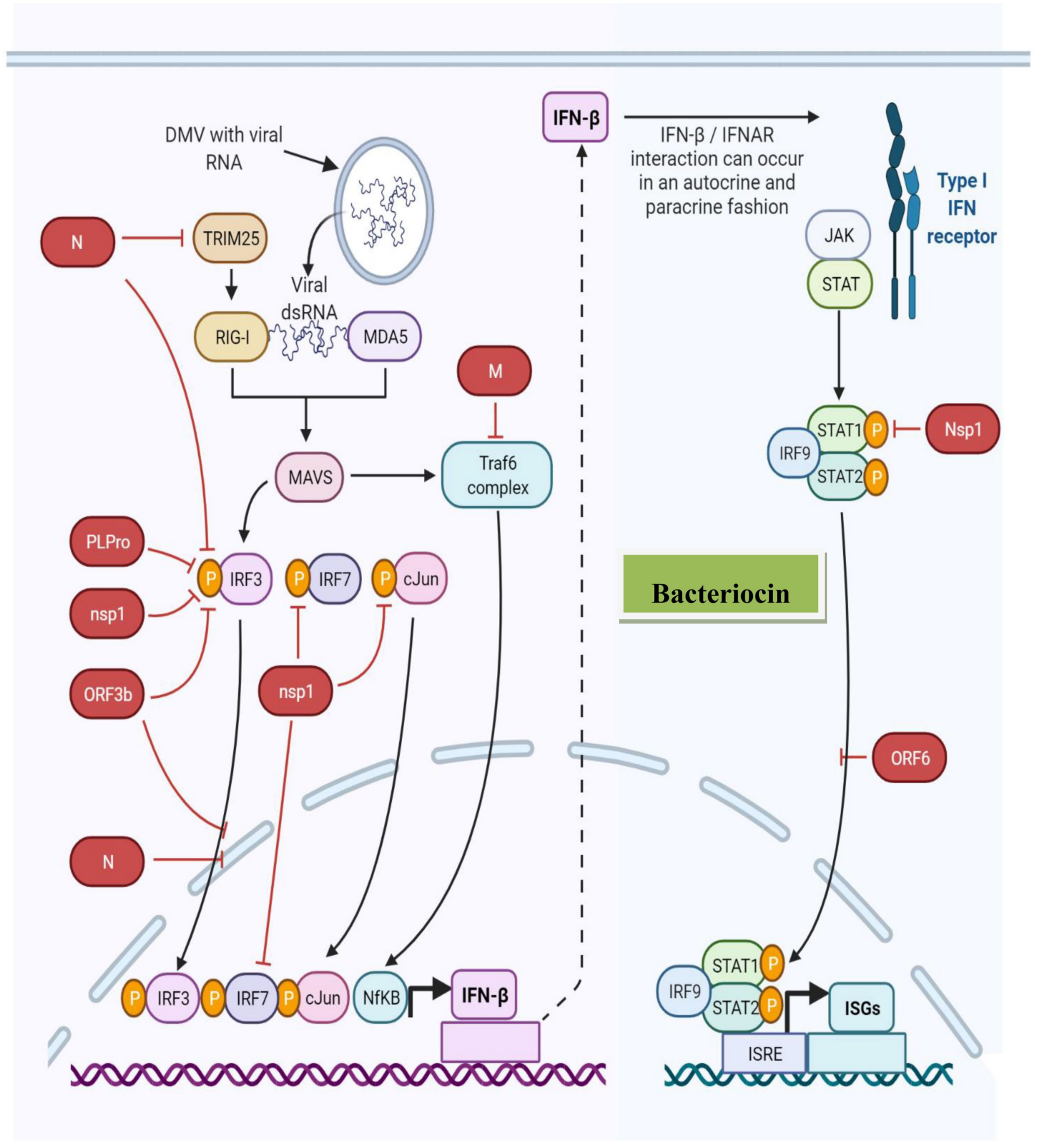
Bacteriocin innate immune antagonism against viral infections.

**Figure 4:**
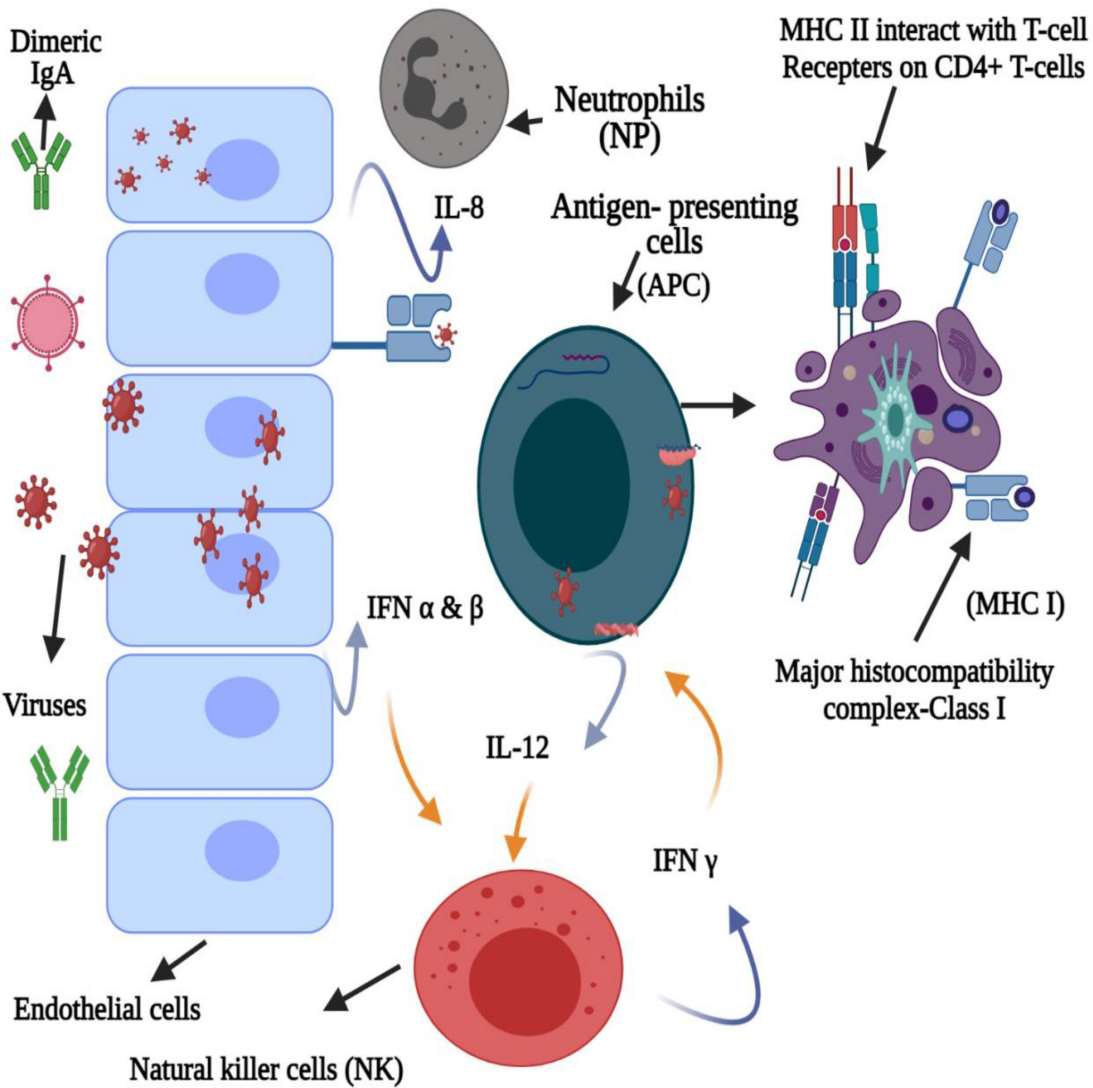
Bacteriocin-induced innate immune response against the virus.

Furthermore, paracrine signaling occurs as a cell interacts with another nearby compartment or cell (unattached with gap junctions), resulting in the diffusion of the produced signaling molecule to a neighboring cell over a short distance. In contrast, autocrine signaling occurs when a cell communicates with the receptors on its own surface (Figure 3). During viral infection, IFN or IFN receptor (IFNR) (IFNAR) association occurs *via* autocrine or paracrine signaling (Silginer et al., 2017). For example, *Lactobacillus* spp. produces bacteriocins involved in IL-12-inducing potentially similar type I IFN production (Alvarez-Vieites et al., 2020; Antushevich, 2020), further demonstrating the immunostimulatory activity of bacteriocins and innate immune antagonistic activity against viral infections. Figure 4 presents the innate immune response to viruses induced by bacteriocins.

The immune system recognizes pathogens in several ways, where inflammasomes induce the production of type I IFNs, IL-1, and IL-18 as the first line of defense against viruses (Dreyer et al., 2019; Antushevich, 2020). Type I IFNs stimulate an antiviral state in the infected host, and cytokines, such as IL, result in inflammation and modulate immune responses, exhibiting antiviral effects (Antushevich, 2020). Furthermore, these bacteriocins elicit an anti-inflammatory response in the innate immune system through dendritic cell signaling, resulting in the release of anti-inflammatory cytokines (i.e., IL-10) (Jenab et al., 2020). Bacteriocins from various studies were shown to increase the CD4 (+) levels and lymphocyte counts and monitor the TNF, IL-6, IL-8, and IL-10 expression levels (Chikindas et al., 2018; Dicks et al., 2018; Ou et al., 2019; Antushevich, 2020; Foysal et al., 2020; Jayawardena et al., 2020; Jenab et al., 2020).

Similarly, Fukuyama et al. (2020) investigated the immunomodulatory properties of bacteriocins and their ability to induce *in vitro* TLR-triggered inflammatory responses. The findings revealed that bacteriocins regulated inflammation and reduced the expression of IL-1α, IL-1β, monocyte chemotactic protein-1, IL-8, and chemokine ligand 3 (CXCL3). It further upregulated the expression of three negative TLR regulators, i.e., the Toll-interacting protein (Tollip), ubiquitin-editing enzyme A20 (TNF-α-induced protein 3, TNFAIP3), and single immunoglobulin IL-1 receptor-related protein (SIGIRR; Mages et al., 2008; Saleh and Trinchieri, 2011; Laiño et al., 2016; Fukuyama et al., 2020).

The potent antiviral activity was mediated by several encoded factors, including IL-2, IL-12, IFN-gamma, TNF-α, CD40 ligand (CD40L), membrane immunoglobulin (mIg), and (cytokine responsive gene 2) Crg-2 (Ramshaw et al., 1997), revealing the mechanism of bacteriocin in combating infection-induced inflammation induced by pathogens. Furthermore, another study reported the bacteriocin inhibitory effect on pathogen-adhesion and invasion of Caco-2 cells and the antiproliferative effects *via* apoptosis induction (Dicks et al., 2018). The authors also showed the reduced levels of TNF-α factor, IL-1β, IL-6, and IL-12 combined with increased levels of IL-10 in serum, further demonstrating the bacteriocin immunomodulatory potential (Jenab et al., 2020).

Another study showed an eight-fold increase in the number of immune cells of probiotic-treated mice, exhibiting significant alterations in the onsite expression of proinflammatory mediators compared with the control group (Chondrou et al., 2020). Similarly, *Lactobacillus shelveticus* produced LZ-R-5 bacteriocin with immunostimulatory activity against viral infection (Ayyash et al., 2020; You et al., 2020). To date, there have been no reported studies on probiotics-derived bacteriocins in preventing and treating COVID-19. However, the phase II experimental research has been used to estimate the effect of an immunomodulatory drug (i.e., the anti-asthma live cell formulation MRx4DP0004) in hospitalized patients with COVID-19. Although the appropriate formulation was not tested, the patients were administered two doses of live cells (4 × 10^9^–4 × 10^10^) for 2 weeks, and the preliminary results were positive. Thus, various probiotics can help control COVID-19 infection and assist as adjuvants for prophylaxis (Dobson et al., 2012; Alvarez-Sieiro et al., 2016).

Additionally, several viruses, such as norovirus (Shinde et al., 2020), rotavirus (RV), or calicivirus (CV), picornaviridae, orthomyxoviridae, paramyxoviridae, reoviridae, and coronaviridae, affect humans (Kamaluddin et al., 2020; Lopez-Santamarina et al., 2021). They are characterized by uncoated RNA, making them exceedingly infectious and fecal transmissible even when the associated infections are easily moderated to last for a short period (Karst, 2016). Regarding the widespread RV vaccination development, norovirus (NV) is the principal cause of severe diarrhea among children and foodborne diseases in developing countries. Approximately 200 million cases were observed among children under the age of 5 years, leading to an estimated 50,000 child deaths per year predominantly in developing countries (Center for Disease Control and Prevention, 2022). Moreover, astroviruses are responsible for 2–9% of all cases of pediatric gastroenteritis globally (Fadhil et al., 2020).

These findings indicated that bacteriocins prevent virus replication more efficiently than viral adsorption; thus, bacteriocins act as novel antiviral agents for viral suppression (Cavicchioli et al., 2018). A large number of studies on the association between specific bacteriocins and antiviral activity showed that probiotics have biological benefits and antiviral properties that regulate and halt the pathogenic virus duplication. Additionally, they are only ascribed to their well-known antibacterial and immune modulation properties. The genesis of microorganisms is also proficient in conducting these activities; it stabilizes the indigenous protective reaction of the ecosystem to a possible territorial invasion, independent of the attack type (viral, bacterial, or fungal) (Cavicchioli et al., 2018).

Based on the available reports, bacteriocins exhibits two mechanisms against viral infection: (i) exhibits antiviral activity before the viral penetration into human cells; (ii) inhibits the viral entry and reduces the cytopathic impact and viral release yield by interacting with the late steps of the viral cycle. The first mechanism of the bacteriocin antiviral activity includes direct contact with viral cells (Al Kassaa et al., 2014), interaction with the epithelial cell surface to influence the electrolyte potential (OlayaGalán et al., 2016), and intracellular inhibition (Serkedjieva et al., 2000). Alternatively, the second mode of action includes the inhibition of the late-stage viral replication (Todorov et al., 2005), blockages on the host cell receptor, repressed intercellular transmission, and modulation of virus immune systems (Drider et al., 2016).

Małaczewska et al. (2019) detected immune modulation by stimulating IL, CD4 (+), and CD8 (+) T cells. Further, Yitbarek et al. (2018) reported that the immune system modulation protects the virus-infected cells. However, the molecular size, hydrophobicity, migration intensity, charge used to cross the epithelial cell membrane, and re-entry ability of tissue cells are the properties of bacteriocins that warrant further exploration (Małaczewska et al., 2019).

Probiotics may be inappropriate for patients unless the pathogenicity and influence of a particular coronavirus on GM has been identified. Besides, the foodborne transmission of COVID-19 is rather uncertain (Li et al., 2021). Therefore, further research should prove that food is a nonprobable agent of viral transmission. It is known that COVID-19 is more susceptible to a weakened immune system. Thus, probiotics as adjuvants or prophylactic and therapeutic agents for COVID-19 treatment should be carefully considered as researchers have reported that probiotics cause numerous septicemias associated with weakened immune systems in those individuals (Koyama et al., 2019).

## Conclusions and Prospects

Probiotics are beneficial microorganisms administered to individuals to improve their healing. In particular, bifidobacteria and LAB probiotics exhibit sustainable therapeutic effects in treating vaginal and GIT viral infections. Moreover, probiotics effectively use cellular components, including peptidoglycans and DNA, and improve the efficacy of the bacterium by producing autoinducing peptides. These emerging probiotics with microbiota-modulation and anti-infection applications are classified into two categories: (i) a first treatment type (prophylaxis) where a low-dose drug is administered daily to maintain the presence of probiotics in the human microbiota; and (ii) a second treatment type where a relatively large dose of medication is administered to the infected individuals, coupled with immunologically sensitive host tissues to treat microbiota dysbiosis. However, the exact mechanism related to the ability of the probiotics to inhibit viral replication remains unknown. Therefore, it is crucial for the scientific and medical communities to focus on beneficial bacteria (probiotics) to combat viral infections.

## Author Contributions

All authors were equal contributors in writing this review article. All authors have read and approved the final manuscript.

## Funding

This review was funded by Khalifa Center for Biotechnology and Genetic Engineering-UAEU (Grant #: 31R286) to SAQ; Abu Dhabi Award for Research Excellence-Department of Education and Knowledge (Grant #: 21S105) to KE-T; and National Key R&D Program of China (2021YFA0910800) and the Special Fund for Development of Strategic Emerging Industries in Shenzhen (JCYJ20190808145613154 and KQJSCX20180328100801771).

## Conflict of Interest

The authors declare that the research was conducted in the absence of any commercial or financial relationships that could be construed as a potential conflict of interest.

## Publisher’s Note

All claims expressed in this article are solely those of the authors and do not necessarily represent those of their affiliated organizations, or those of the publisher, the editors and the reviewers. Any product that may be evaluated in this article, or claim that may be made by its manufacturer, is not guaranteed or endorsed by the publisher.
